# Parameterization and validation of an ungulate‐pasture model

**DOI:** 10.1002/ece3.3358

**Published:** 2017-09-07

**Authors:** Antti‐Juhani Pekkarinen, Jouko Kumpula, Olli Tahvonen

**Affiliations:** ^1^ Natural Resources Institute Finland Helsinki Finland; ^2^ Department of Forest Sciences University of Helsinki Helsinki Finland; ^3^ Natural Resources Institute Finland Kaamanen Finland

**Keywords:** herbivore management, lichen, reindeer, trampling, ungulate, wastage

## Abstract

Ungulate grazing and trampling strongly affect pastures and ecosystems throughout the world. Ecological population models are used for studying these systems and determining the guidelines for sustainable and economically viable management. However, the effect of trampling and other resource wastage is either not taken into account or quantified with data in earlier models. Also, the ability of models to describe the herbivore impact on pastures is usually not validated. We used a detailed model and data to study the level of winter‐ and summertime lichen wastage by reindeer and the effects of wastage on population sizes and management. We also validated the model with respect to its ability of predicting changes in lichen biomass and compared the actual management in herding districts with model results. The modeling efficiency value (0.75) and visual comparison between the model predictions and data showed that the model was able to describe the changes in lichen pastures caused by reindeer grazing and trampling. At the current lichen biomass levels in the northernmost Finland, the lichen wastage varied from 0 to 1 times the lichen intake during winter and from 6 to 10 times the intake during summer. With a higher value for wastage, reindeer numbers and net revenues were lower in the economically optimal solutions. Higher wastage also favored the use of supplementary feeding in the optimal steady state. Actual reindeer numbers in the districts were higher than in the optimal steady‐state solutions for the model in 18 herding districts out of 20. *Synthesis and applications*. We show that a complex model can be used for analyzing ungulate‐pasture dynamics and sustainable management if the model is parameterized and validated for the system. Wastage levels caused by trampling and other causes should be quantified with data as they strongly affect the results and management recommendations. Summertime lichen wastage caused by reindeer is higher than expected, which suggests that seasonal pasture rotation should be used to prevent the heavy trampling of winter lichen pastures during summer. In the present situation, reindeer numbers in northernmost Finland are in most cases higher than in the management solutions given by the model.

## 1 INTRODUCTION

Throughout the world, large mammalian herbivores strongly affect their pastures and can have significant effects on entire ecosystems (Hobbs, 1996). Herbivores directly affect plant species composition and diversity by grazing or browsing and indirectly by trampling, urination, and defecation (Augustine & McNaughton, 1998; Hobbs, 1996). Herbivore management is often very intensive, especially with ungulates that have high economic value (Gordon, Hester, & Festa‐Bianchet, 2004). All ungulate‐pasture systems are under direct or indirect human influence (Augustine & McNaughton, 1998), which can deviate the system far from its natural state and sometimes lead to overgrazing (Mysterud, 2006). The introduction of new herbivore species, predator removal, and supplementary feeding can all cause a situation where herd size increases to a very high level, which strongly affects the resource species and ecosystem in question (Frank, McNaughton, & Tracy, 1998). One of the main tools in managing ecosystems is to set animal densities at desired levels, and therefore, understanding the relation between ungulate populations and plant communities is crucial from a management perspective (Augustine & McNaughton, 1998). As ungulate systems affect large areas over a long time scale it is often too costly to use empirical field experiments for studying ungulate‐plant dynamics. However, ecological population models are apt methods for studying these systems.

Ecological models are common when studying population dynamics, the outcomes of different management actions, and the optimal use of natural resources. One of the central objectives in applied ecological research is to provide objective information that ecosystem managers can use to achieve economic, conservation, and environmental goals (Gordon et al., 2004). Simple models can provide understanding of the qualitative dynamics in herbivore–plant interaction, but we need more complex models with a firm empirical background to have sufficient quantitative solutions and predictions. Validation and parameter estimation are necessary steps in ensuring that model results are suitable for this purpose (Mayer and Butler 1993). Complex system models are difficult to validate, as data acquisition is often time‐consuming and costly (Brown & Kulasiri, 1996). For the same reasons detailed models for mammalian herbivores often lack validation and empirically based estimation of some key parameters (Sleep & Loehle, 2010). In our study, we use detailed data from northernmost Finland to parametrize and validate a complex reindeer‐lichen model. We then use the validated model with estimated parameters to study the solutions for optimal management and compare them with previous research and empirical data from the herding districts.

One key task in ecology is to study how plants and ecosystems are affected by high herbivore densities (Hansen, Henriksen, Aanes, & Sæther, 2007). Herbivore–plant models are used for this purpose, as they focus on the interaction between herbivores and their resource species. Herbivores reduce plant biomass by consuming it for energy and by wasting it, for example by trampling. In classical biomass models for predator–prey interaction, the conversion efficiency parameter describes how efficiently the depletion of prey converts into the offspring or biomass growth of predators. This conversion efficiency parameter can be interpreted to account for the inefficiency in converting an ingested resource into new biomass along with the wastage of the resource. In complex herbivore–plant models, the conversion of a resource to new offspring (and growth) is described through mechanisms that usually include at least the energy composition of the resource and the energy budget of the herbivore, from which growth, mortality, and reproduction are computed. These processes are described in great detail and are based on vast empirical research. However, resource wastage is not often taken into account or is described with less empirical basis and detail.

Oene et al. (1999) developed a model of plant–herbivore interaction and described the wastage of plant species by herbivores as a fraction of the food intake. Wastages by cattle used in Oene et al. (1999) varied from 0.1 to 4 depending on plant species, and thus, the results were heavily influenced by this parameter. Gaare and Skogland (1979) described the lichen wastage relative to intake rate as a function of lichen biomass in their elementary reindeer‐lichen model. Based on preliminary data, they suggested that lichen wastage varies between 2 and 10 times the intake rate. Moxnes, Danell, Gaare, and Kumpula (2001) also described wastage as a function of lichen biomass in their bioeconomic reindeer‐pasture model, but the wastage level was substantially lower than that used by Gaare and Skogland (1979) and varied from 0.5 to 4.5 times the intake rate. Moxnes et al. (2001) found that lichen biomass in an optimal steady state is lower than the biomass that maximizes yearly lichen growth. The main reason for this was that the wastage relative to the intake rate was assumed to be a function of lichen biomass. Tahvonen, Kumpula, and Pekkarinen (2014) used a constant relative wastage of 0.3 times the wintertime intake rate, and Pekkarinen, Kumpula, and Tahvonen (2015) included wastages also for the spring, summer, and autumn seasons, which were 0.6, 2, and 0.6, respectively. They found that at the same quantity of available lichen pastures the annual net revenues and optimal herd size are lower without a pasture rotation system. This is because without pasture rotation lichen pastures are highly degraded during summer, which is mainly caused by trampling.

As seen above, the estimates for lichen wastage vary substantially, and despite the wastage strongly affecting the results of previous models it has not been quantified with empirical data. In our study, we compute the first data‐based estimate of this critical wastage parameter using detailed data from the 20 northernmost reindeer herding districts of Finland. We then use the estimated parameter value in the validation of an age‐ and sex‐structured reindeer‐lichen model. The validation and parameter estimations are performed to study how well the model can describe changes in lichen biomass when reindeer numbers, management practises, and pasture types are known. We use the validated model to study how the new estimated lichen wastage changes the solutions for economically viable reindeer husbandry. Finally, we use the model to evaluate the current situation of the reindeer‐lichen system in northernmost Finland.

## 2 DATA AND METHOD

We use a model published by Tahvonen et al. (2014) and Pekkarinen et al. (2015). It describes an age‐ and sex‐structured reindeer population and the dynamics between the reindeer population and lichen pastures. It also includes the effects of arboreal lichen pastures, supplementary feeding, and the pasture rotation system. The model includes 17 female and 13 male age classes and a detailed description of winter energy resource utilization by the reindeer population. Reproduction in the model is specified by a modified harmonic mean mating system, and the diet choice between various winter energy resources follows the principles of the optimal foraging theory. Wintertime energy intake defines an individual's overwinter weight decrease and its consequences on mortality and reproduction. Lichen growth in the model depends on habitat type and lichen biomass after consumption. We use the model formulation presented in Pekkarinen et al. (2015), and the parameter values describing the pasture conditions, management practises, reindeer numbers, and the mortality due to traffic and predators are from the actual data for northernmost Finland (Table 1, Appendices 1 and 2). In addition, the description and parameters for lichen wastage are estimated in our study.

**Table 1:** Parameter values for pasture conditions in the 20 northernmost herding districts in Finland. Descriptions of the symbols and terms: HD: number for herding district (not the official number), Feeding: supplementary food (kg) per reindeer per winter, *A*: area (ha) of lichen pastures, *A*_Q_: area (ha) of arboreal lichen pastures, *q*: biomass (kg/ha) of the arboreal lichens available for reindeer, *z*_1995_: lichen biomass (kg/ha) in 1995, *z*_2008_: lichen biomass (kg/ha) in 2008, *g*: growth rate of lichen compared to the growth rate in mature/old pine forests, H.metal: decrease (%) in lichen growth due to heavy metal accumulation, Infrastr.: the coverage and disturbance area of infrastructure (%)

HD	Pasture rotation^a^	Feeding^b^	*A* c	*A*_Q_ d	*q* e	*z*_1995_ c	*z*_2008_ c	*g* f	H.metal^g^	Infrastr.^h^
1	NO	12	78,780	1,836	6	33	129	0.49	20	3.4
2	NO	12	58,672	1,830	6	65	76	0.57	20	4.7
3	YES	12	16,542	10,169	6	801	244	0.72	40	5.2
4	YES	75	21,537	17,051	6	1,606	409	0.74	20	9.8
5	YES	12	11,064	20,168	6	1,446	348	0.85	60	5.4
6	NO	12	12,043	15,976	6	1,877	464	0.91	40	2.6
7	NO	12	72,330	43,599	9	470	313	0.78	20	14.8
8	NO	12	67,088	35,238	9	1,021	603	0.77	20	7.5
9	YES	12	32,940	26,491	9	594	421	0.66	–	2.5
10	YES	12	23,328	23,860	6	431	252	0.73	20	3.8
11	NO	12	82,343	16,444	6	409	128	0.62	–	4.8
12	NO	12	113,197	3,332	6	364	246	0.50	–	4.8
13	NO	75	66,937	39,235	12	187	184	0.82	–	27.2
14	YES	37	9,841	17,016	12	501	324	0.70	–	10
15	NO	200	42,062	41,494	12	332	202	0.77	–	10.6
16	YES	125	12,546	24,511	12	365	194	0.76	–	10.6
17	NO	200	38,345	34,047	12	421	102	0.75	–	13
18	YES	75	57,930	56,927	9	600^i^	820^i^	0.78	–	4.3
19	NO	75	131,049	83,908	12	563	366	0.75	–	4.8
20	NO	12	44,281	28,528	12	237	250	0.72	–	8.4

a Kumpula et al. (2009).

b Nieminen et al. (2006).

c Kumpula et al. (2009).

d Kumpula et al. (2009) assuming that only 40% of arboreal lichen pastures have sufficient biomass.

e Estimate based on Kumpula et al. (2009).

f Data from Kumpula et al. (2014), formula from Pekkarinen et al. (2015).

g Estimated effects on lichen growth rate based on heavy metal concentrations measured in mosses (Metla 2012).

h Kumpula et al. (2014).

i *z*_2008_ includes measurements from additional sites. *z*_1995_ is estimated combining field observations and measurements.

### 2.1 Data from the herding districts

Parameter values for the pasture conditions and reindeer herding practises are shown in Table 1. The data describes the 20 northernmost herding districts in Finland from 1995 to 2008. Lichen biomass has been estimated in 1995–1996 for the first time and in 2005–2008 for the second time on the same sites in all 20 herding districts (Kumpula et al., 2009). However, approximately 70% of the sites in herding district number 18, measured both in 1995–1996 and 2005–2008, were not under reindeer grazing. Thus, for this district we also include measurements from additional sites made in 2005–2008 and combine the biomass measurements with the field observations made in the first pasture inventory (Kumpula, Colpaert, Kumpula, & Nieminen, 1997). Lichen biomasses for all the herding districts with pasture rotation are computed using only the sites that are located inside the winter pastures areas.

Data for the lichen pasture sizes are from Kumpula et al. (2009). We divide the districts into two groups depending on whether or not pasture rotation was used between 1995 and 2008. If pasture rotation was not used we assume that all lichen pastures in that district are under consumption during the entire year. For the districts with pasture rotation, we assume that the lichen pastures located in the winter grazing area are annually grazed for 6 months during the winter period and for 4 weeks during the spring period. The 4 weeks during spring are included because in the study districts reindeer usually still stay in their winter pastures also during early spring or can go already in late autumn.

Arboreal lichen availabilities are estimated from Kumpula et al. (2009) and divided into three categories (high availability = 12 kg/ha, medium availability = 9 kg/ha, low availability = 6 kg/ha). We estimated that in only 40% of the arboreal lichen pastures reported in Kumpula et al. (2009) availability was high enough (>6 kg/ha) to offer a significant energy resource for reindeer. We use the estimate by Nieminen, Maijala, and Soveri (2006) for the amount of supplementary feeding used in each district.

Lechowicz (1982) showed that nickel and other air deposited heavy metals can decrease the growth rate of lichen. Kumpula, Kurkilahti, Helle, and Colpaert (2014) also found that the heavy metal deposits transported by air from metal foundries affected the lichen biomass and probably also their regeneration rate. We take this into account in our model by decreasing the growth rate of lichens for herding districts where high nickel and copper concentrations have been observed in moss samples (Metla 2012). We assume that in herding district 5, where the measured nickel concentration is more than 15 mg/kg, lichen growth is reduced by 60% because of the effect by heavy metals accumulation. In herding districts 3 and 6, the concentration is 9–12 mg/kg, and the growth rate reduction is 40%. In districts where the concentration is 3–6 mg/kg; the growth rate reduction is 20%. We performed a sensitivity analysis by decreasing and increasing the effect of heavy metal deposits by 50% and also by computing the validation and parameter estimation results without the effect of heavy metals.

Kumpula et al. (2014) also showed that the total coverage and disturbance area of infrastructure affected the lichen biomass on pastures. They suggested that infrastructure and disturbances might increase the effect of reindeer on lichens by increasing movements, trampling, and temporal grazing pressure. We take this into account by increasing the total lichen wastage with infrastructure‐induced wastage. We describe this infrastructure‐induced wastage as a function of the area of infrastructure‐associated disturbance in the study districts. We assume that when 25% of the area of a herding district is under a disturbance, the infrastructure‐induced wastage is 1. Thus, the value of infrastructure‐induced wastage varies between 0.1 and 1.088 depending on herding district. We performed a sensitivity analysis on infrastructure‐induced wastage by decreasing and increasing the effect of this wastage factor by 50% and also by computing the results without the effect of infrastructure.

### 2.2 Data for reindeer numbers and mortality

The data for reindeer numbers and mortality for 1995–2007 are from the Reindeer Herders’ Association (Appendices 1 and 2). The number of annually slaughtered calves, adult females and adult males, is known, and also the number of calves, adult females and adult males, left alive is counted each autumn. However, some part of the reindeer population always remains uncounted. By comparing the number of reindeer reportedly left alive after slaughtering with the number of reindeer (over 1 year old) counted and slaughtered during the next autumn, and taking into account wintertime reindeer mortality, we can compute the minimum number of uncounted reindeer in the previous autumn. The number of uncounted reindeer calculated in this way varied highly between the years and herding districts (Appendix 2). If part of the population remains uncounted for two or more years the individuals do not show up in the data until the year they are once again counted. Thus, we can only calculate the minimum number of uncounted reindeer, and the true number must be clearly higher. Therefore, we assume that the average share of uncounted reindeer is 10% in all herding districts. However, when the data from the herding districts (Appendix 2) show that more than 10% of reindeer were uncounted during some year, we then use that value for the given year to correct the average estimate. We also assume that the share of uncounted reindeer is equal between all age and sex classes.

The mortality data from traffic accidents are assumed to be precise, but the total number of reindeer lost due to predation is not well known. When the Finnish government pays compensations for predation (Finlex 2009), it estimates that one‐third of the dead reindeer are not found. In our study, we use this same estimate and assume that the true number of adult reindeer dead because of predation is 1.5 times the number of reindeer reported dead due the predation. In our data, the yearly variation within the reindeer herding districts was relatively small; thus, we use the long‐term average mortalities, which varied from 1% to 6% between herding districts (Appendix 2). The number of predated calves cannot be derived from the mortality data, because the predation on calves takes place mainly during the summer season when it is difficult to find dead calves because they are often completely consumed by predators. However, for parameter estimation and model validation, we can directly use the number of calves reported in autumn. Estimates for calf summer mortality are needed only when we compute the management solutions for the model with data from the herding districts. For that calculation, we assume that calf mortality is 10%.

### 2.3 Cross‐validation for parameter estimation and model validation

Model validation must be performed using data that have not been used for building the model or in parameter estimation (Fielding & Bell, 1997). However, one problem with this is that it is often too difficult or costly to acquire new data for the validation. In many studies, sample splitting is used as a simple solution, and the data are split between “a calibration sample” and “a test sample.” However, using this method means that half of the valuable data are lost for both parameter estimation and model validation, when using small‐sized data. This problem can be solved using leave‐one‐out cross‐validation (also known as jackknife sampling) (Fielding & Bell, 1997; Hawkins, Basak, & Mills, 2003). In this method, each data point in a sample (sample size = *n*) is removed in turn, and the calibration is performed using the rest of the data (*n* − 1). For model validation, the calibrated model is next used for predicting the single value that is compared with the value left out of the calibration sample. This procedure is repeated *n* times, and each data point is removed in turn. Thus, each data point can be used for validation and parameter estimation, and the model is calibrated for each validation computation without utilizing the data used in the validation.

We use this cross‐validation method for estimating lichen wastage and for validating the model's ability to predict lichen biomass change. Data for each herding district are left out in turn, and the parameter estimation is performed using the remaining 19 herding districts. We then used the model and estimated a wastage parameter to predict the lichen biomass change in the herding district that was left out from the parameter estimation. This is repeated for all 20 districts. Thus, for each herding district, the wastage parameter used in the model validation is estimated without using data from that district.

### 2.4 Comparing model and data with modeling efficiency

Both model validation and parameter estimation require comparisons between model predictions and observed data. A comparison can be made using subjective graphical visualizations and/or objective statistical techniques. Both are useful, but parameter estimation automatization using computers requires statistical techniques. As a visual comparison we use observed vs. predicted plots recommended by Mayer and Butler (1993). For statistical comparison, we use modeling efficiency, which is suggested as a best overall measure of agreement between observed and simulated values (Mayer & Butler, 1993). It is a dimensionless statistic that directly relates model predictions to observed data and is defined (in the framework of this study) as:

(1) $$\text{EF}\;=\;1-\frac{\sum_{\mathrm{HD}\;=\;1}^{n}{\left(z_{d,\mathrm{HD}}-z_{m,\mathrm{HD}}\right)}^{2}}{\sum_{\mathrm{HD}=1}^{n}{\left(z_{d,\mathrm{HD}}-z_{d,a}\right)}^{2}},\text{HD}\;=\;1\ldots 20,$$

where *z*_d,HD_ denotes the measured lichen biomass for 2008 in a herding district HD, and *z*_m,HD_ denotes the model prediction for lichen biomass in herding district HD for 2008. HD is the number of herding district, and *z*_d,a_ is the average biomass in 2008 for all herding districts. We additionally compute the Pearson product–moment correlation coefficient as a description of the correlation between model predictions and the data.

### 2.5 Estimating parameters for lichen wastage

In our study, we estimate the parameters for lichen wastage using a model and detailed data. All other model parameters are predetermined based on existing research and data. The predetermined parameters are presented in Table 1, Appendices 1 and 2, and Pekkarinen et al. (2015). The level of effects caused by heavy metal accumulation and infrastructure‐induced wastage is based on raw estimates, and sensitivity analyzes are therefore performed for both. Wastage is the part of total consumption, that is not used for energy and we denote it by *W*_e_, *e* = wi, sp, su, au, where wi, sp, su, au denote the winter, spring, summer, and autumn seasons, respectively. Because our data are not sufficient to differentiate spring, summer, and autumn wastages from each other, we assume that spring and autumn wastages are half of the summer wastage (*W*_sp_ = *W*_au_ = 0.5*W*_su_). In parameter estimation, this assumption only affects the relative wastages between the spring, summer, and autumn seasons, but not the relation between winter wastage and total wastage outside wintertime.

In Pekkarinen et al. (2015), the wastage is described as a constant share of total consumption. However, Gaare and Skogland (1979) and Moxnes et al. (2001) define the wastage relative to intake rate as a function of lichen biomass. We test whether or not the wastage relative to intake rate is better described by a function of lichen biomass or by a constant. We use a linear function, as it is the simplest way to describe wastage relative to intake rate as a function of lichen biomass:

(2) $$W_{e}\;=\;p_{e,1}\;+\;\left(p_{e,2}-p_{e,1}\right)z_{t}/1,000,\;e\;=\;\text{wi,sp,su,au.}$$

Parameters *p*_e1_ and *p*_e2_ indicate the wastages when lichen biomass is 0 and 1,000 kg/ha, and *z*_*t*_ is the lichen biomass in year *t*.

Parameter estimation can be performed by minimizing the loss function or maximizing the probability of observing the experimental data (Jaqaman and Danuser 2006). In our study, we maximize the modeling efficiency to find the wastage parameters for winter (*p*_wi,1_, *p*_wi,2_) and summer (*p*_su,1_, *p*_su,2_) that give the best fit between the model and data. The objective was given as:

(3) $$\max_{\left\{p_{\mathrm{wi},1},p_{\mathrm{wi},2},p_{\mathrm{su},1},p_{\mathrm{su},2}\right\}}\text{EF}\;=\;1-\frac{\sum_{\mathrm{HD}=1}^{n}{\left(z_{d,\mathrm{HD}}-z_{m,\mathrm{HD}}\right)}^{2}}{\sum_{\mathrm{HD}=1}^{n}{\left(z_{d,\mathrm{HD}}-z_{d,a}\right)}^{2}},\text{HD}\;=\;1\ldots 19.$$

The total number of herding districts used in our study is 20, but during parameter estimation with cross‐validation each of the districts is taken out in turn, and therefore, the number of districts in each estimation is 19. We use the estimated wastage parameters for model validation, but as we use cross‐validation, the parameters are estimated separately for each district without using data from that district. Thus, we have not used the calibration data from the district in which the prediction of the lichen biomass is performed in the model validation. However, as the same dataset is used for both parameter estimation and model validation, the data points are not totally independent of each other temporally and spatially. For example, our data describe the time period from 1995 to 2008, and thus, general weather conditions during these years may have an effect of the solutions.

When the wastage relative to the intake rate is constant it can be described with a single parameter for each season (i.e., *p*_wi,1_ = *p*_wi,2_ and *p*_su,1_ = *p*_su,2_). We call this case constant wastage. But when wastage relative to intake is described as a linear function of the lichen biomass (i.e., *p*_wi,1_ ≠* p*_wi,2_ and *p*_su,1_ ≠* p*_su,2_), we need two parameters for both winter (*p*_wi,1_, *p*_wi,2_) and summer (*p*_su,1_, *p*_su,2_). This we call linear wastage. For both linear and constant wastage, the maximization is subject to the model presented in Pekkarinen et al. (2015) with values from Table 1, Appendices 1 and 2.

### 2.6 Economic optimization

We compute the optimization results the same way as described in Pekkarinen et al. (2015) using both constant and linear wastage. We also compare the optimal solutions with the data from the 20 northernmost herding districts in Finland. As we have used cross‐validation in parameter estimation, we have 20 different values for each estimated parameter. In economic optimization, we use the average values of the estimated wastage parameters. The objective function is identical with that presented in Pekkarinen et al. (2015):

(4) $$\max_{\left\{h_{s,t}^{i},v_{t}^{k},t=0,1,\ldots ,i=f,m,\;s=0,\cdots ,n_{i},k=a,b\right\}}J\;=\;\sum_{t=0}^{\infty }{\left(R_{t}-C_{t}\right)}^{\alpha }{\left(\frac{1}{1+r}\right)}^{t}.$$

The objective is subject to the model presented in Pekkarinen et al. (2015) and equation (2) from this study with parameter values from the parameter estimation. When optimization is performed using values from a herding district the parameters are taken from Table 1, Appendices 1 and 2. For all optimizations, we apply Knitro (version 7.0.0) optimization software that applies powerful gradient‐based interior point algorithms (Byrd, Nocedal, & Waltz, 2006).

## 3 RESULTS

### 3.1 Parameter estimation

**Figure 1:**
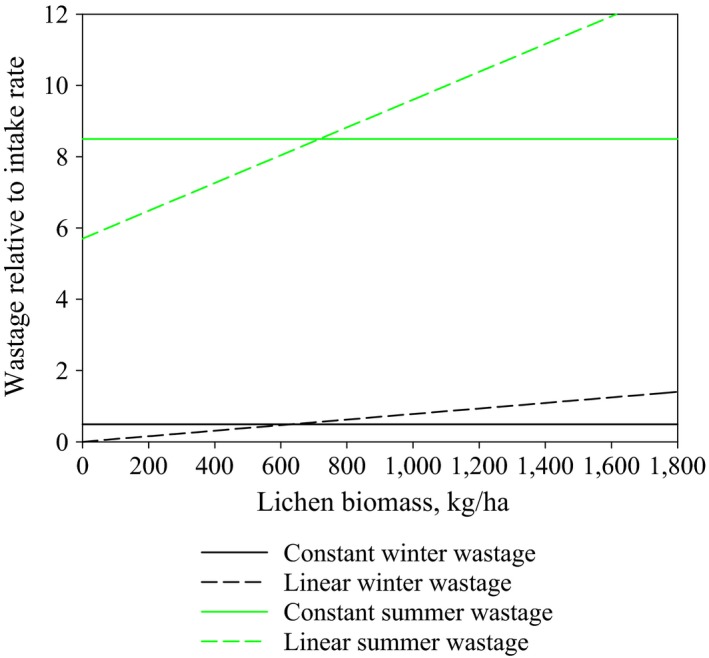
Estimated average lichen wastages relative to the intake rate of lichen during winter. Results for constant and linear wastages for the winter and summer periods

We first estimated the best‐fitting values for lichen wastage during winter and summer assuming that the relative wastage is not dependent on lichen biomass (constant wastage). Parameter estimation was performed 20 times, excluding each herding district in turn and optimizing the values for lichen wastage during winter and summer. Results are shown in Figure 1. The average wastage was 8.5 during summer and 0.5 during winter. Wastage for spring and autumn seasons was assumed to be half of the summer wastage.

We then estimated the parameter values for the lichen wastage equations (equation (2)) assuming that the wastage relative to intake rate is linearly dependent on lichen biomass (linear wastage). Parameter estimation was again performed 20 times, excluding each herding district in turn. Results for the average wastages are shown in Figure 1 (dashed lines). When lichen biomass reaches 0 kg/ha; the average wastage value was 5.7 for the summer season and 0 for winter. At a lichen biomass level of 1,000 kg/ha, the wastages were 9.6 and 0.7 for summer and winter, respectively. Our data points cover lichen biomasses from 65 to 1,800 kg/ha (see Table 1). Evaluating the wastage for higher lichen biomasses was not possible using our data.

### 3.2 Model validation

**Figure 2:**
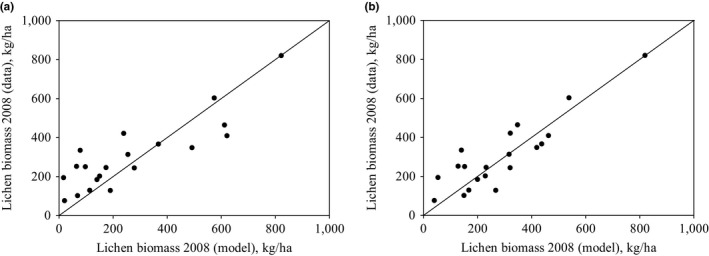
Observed vs. predicted lichen biomasses for year 2008 for constant (a) and linear (b) wastage. The line presents the perfect fit between model results and data. The Pearson product–moment correlation coefficient is 0.864 (*p* = 8.99 × 10^−8^) for constant wastage and 0.883 (*p* = 2.46 × 10^−8^) for linear wastage

**Figure 3:**
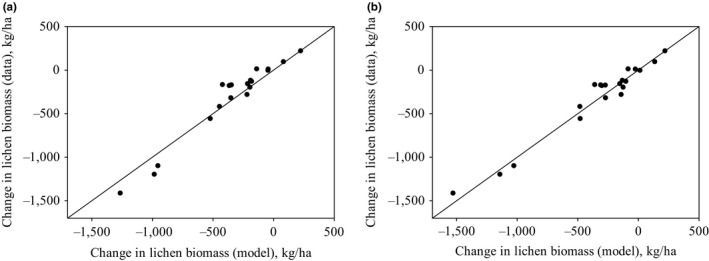
Observed vs. predicted change in lichen biomass from 1995 to 2008 for constant (a) and linear (b) wastage. The line presents the perfect fit between model results and data. The Pearson product–moment correlation coefficient is 0.972 (*p* = 9.94 × 10^−13^) for constant wastage and 0.98 (*p* = 5.53 × 10^−14^) for linear wastage

We first tested model validity using constant values for wastage. Visual observation (Figures 2a and 3a) and modeling efficiency (EF = 0.52) showed that if the wastage relative to intake rate is constant, the model can describe the changes in lichen biomass relatively well. However, the model tends to predict too low lichen biomasses when the measured biomass is low (less than 300 kg/ha). We then tested the model with the estimated linear function for lichen wastage. The model very efficiently predicted both the change in lichen biomass (Figure 2b) and the direction of the change (Figure 3b) (modeling efficiency was 0.75). The Pearson product–moment correlation coefficient was high for models with both wastage functions, which show clear correlation between model predictions and the data.

### 3.3 Comparing model versions

The study by Pekkarinen et al. (2015) extends the model by Tahvonen et al. (2014) by including pasture rotation, supplementary feeding, arboreal lichen pastures, and various growth rates for ground lichen. We additionally included the effects of heavy metal deposits and infrastructure‐associated disturbance in our study. Table 2 shows how modeling efficiency is affected if the extensions are excluded from the model one at a time. Whether or not feeding is included into a model does not affect the model fit, but excluding arboreal lichens or the variation in growth rates of ground lichen slightly decreases modeling efficiency. Excluding all of these three extensions decreases modeling efficiency from 0.75 to 0.54. If the pasture rotation system is not taken into account the model fit decreases significantly and modeling efficiency drops to 0.04. Sensitivity analyzes on both the effects of nickel and infrastructure showed that including them increases modeling efficiency. Finally, we tested model performance without any extensions, first assuming that reindeer are allowed to graze freely year‐round in all districts, and then assuming that a seasonal pasture rotation system is in use in all districts. The latter model is similar to the one we analyzed in Tahvonen et al. (2014). The modeling efficiencies for these cases are −0.4 and −0.53 respectively, showing that despite optimized wastage parameters, the model cannot predict the changes in lichen biomass in various herding districts without the relevant extensions presented in Pekkarinen et al. (2015) and in our study.

**Table 2:** Values for modeling efficiency for various representations of the model. Corresponding extensions are excluded from the full model (linear wastage) one at a time

Version	Modeling efficiency
Full model (linear wastage)	0.75
Full model (constant wastage)	0.52
No feeding	0.75
No arboreal lichens	0.71
One growth rate	0.67
None above	0.54
No pasture rotation	0.04
No infrastructure	0.69
50% infra	0.74
150% infra	0.72
No nickel	0.51
50% nickel	0.68
150% nickel	0.55
No extensions	
No pasture rotation	−0.40
Pasture rotation in all districts^a^	−0.53

a Corresponding to the model in Tahvonen et al. (2014).

### 3.4 Economic optimization

**Figure 4:**
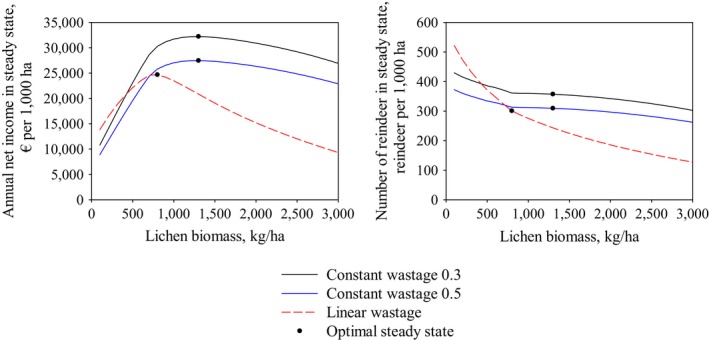
Yearly net incomes and reindeer population sizes with their optimal points in steady states corresponding to various lichen biomasses. The wastage relative to the intake rate is constant in the solutions represented by the solid lines. In the case of a dashed line, the relative wastage is a linear function of lichen biomass

Figure 4 shows the steady‐state solutions with a 0% interest rate using three different wastage functions. Solutions represent a fictional herding district with no arboreal lichen, heavy metal accumulation, or infrastructure, and where all lichen pastures are in old or mature pine forests and a closed pasture rotation system is used. We maximized the yearly net income for each steady state corresponding to lichen biomasses from 100 to 6,000 kg/ha. With all present wastage functions, the steady‐state income increases as lichen biomass increases from 100 kg/ha until the optimal steady state is reached (1,280 kg/ha for constant wastage and 750 kg/ha for linear wastage). Increasing the lichen biomass beyond these optimal steady states decreases yearly net income. Increasing the constant wastage from 0.3 used in Pekkarinen et al. (2015) to 0.5 decreases the optimal steady‐state net income and optimal size of the reindeer population but not the optimal lichen biomass (1,280 kg/ha). The optimal lichen biomass level is lower with the linear wastage function.

**Figure 5:**
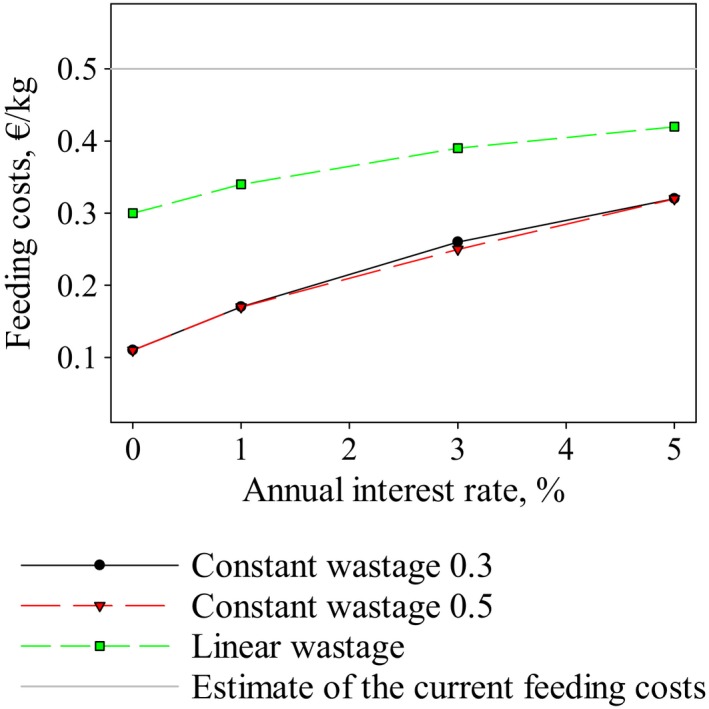
Interest rate effect on the use of supplementary feeding in the optimal steady states. Curves represent the feeding costs for which it is optimal to offer supplementary food as a main winter energy resource for reindeer, resulting in very low lichen densities. Feeding is not used in situations located above the curves, and reindeer herding is based on natural pastures. Intensive supplementary feeding is used below the curves, leading to very low lichen densities. Results are computed for a district with closed pasture rotation, a high lichen growth rate and no arboreal lichen pastures

Figure 5 shows the effect of interest rate and feeding costs on the choice of using supplementary feeding in the optimal steady state. Changing the constant wastage level from 0.3 to 0.5 does not affect whether or not supplementary feeding is used in the optimal steady state. However, with the linear wastage function, supplementary feeding is also used in the optimal steady states with higher feeding costs.

### 3.5 Comparing optimization results with data

**Figure 6:**
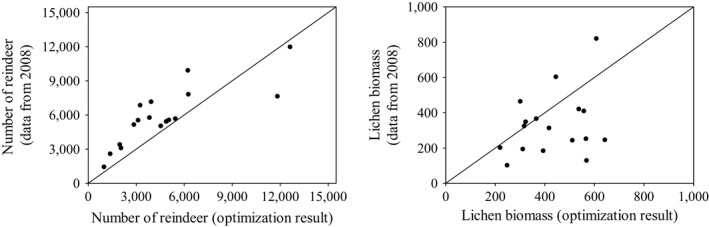
Optimal solutions for the model using data from the herding districts compared with reindeer numbers and lichen biomass from the herding districts data in 2008. The line presents the perfect fit between model results and data

Figure 6 shows the comparison between the optimal steady‐state solutions and the data. The model solutions are computed with a linear wastage function, a 2% interest rate and using data from Table 1 (excluding lichen biomasses) for each of the herding districts. In all except two herding districts, the actual number of reindeer in 2008 was higher than in the optimal model solution (Figure 6a). Lichen biomass has clearly increased from 1995 to 2008 in the only herding district where reindeer numbers were much lower compared to the optimal solution. In all other districts, the lichen biomass has decreased or increased only slightly. Figure 6b shows that in the present situation the lichen biomasses in optimal solutions vary between 200 and 700 kg/ha depending on the herding district. The biomass range in the data is also relatively similar, varying from 100 to 800 kg/ha. However, the biomass in 2008 was lower than the biomass from the optimal model solution in more than half of the districts.

## 4 DISCUSSION

**Figure 7:**
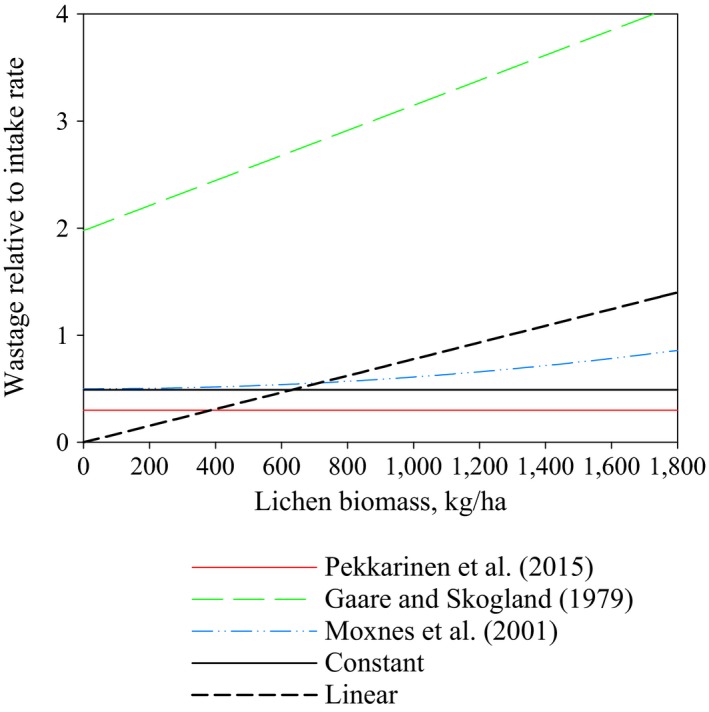
Lichen wastage relative to intake rate during winter. Wastages from previous research along with results for the constant and linear wastages found in our study are reported

According to our results, when lichen biomass is below 1,000 kg/ha, the best fit between model predictions and data is found when the wastage during winter is between 0 and 0.7 times the intake rate. As our method assumes that the reduction in lichen biomass from 1995 to 2008 depends only on factors taken into account in the model, it is possible that the true wastages are somewhat smaller than those reported in our study. This would be the case if, for example, tourism would have a large enough effect on lichen biomass relative to the effect by reindeer. However, reindeer‐related processes are generally seen as the primary factors affecting the reduction in lichen biomass (Kumpula et al., 2014). Figure 7 shows that the wastage values used in previous bioeconomic optimization studies by Moxnes et al. (2001), Tahvonen et al. (2014),and Pekkarinen et al. (2015) are all within this range. However, wastages used in Gaare and Skogland (1979) are much higher. They reported that based on preliminary data the wastage is twice the intake when lichen biomass is at the lowest utilizable level and 10 times the intake when lichen biomass is at carrying capacity. According to our results, these values are too high to be used for wintertime. However, our results indicate that wastage mainly due to trampling is much higher in the summer season than in winter. Thus, wastages described by Gaare and Skogland (1979) might better describe the average wastage throughout the entire year. In fact, our results show the average wastage for the entire year when using the linear wastage function to be 2.5 at the minimum lichen biomass and 4.4 at a lichen biomass of 1,000 kg/ha. These values are close to the results by Gaare and Skogland (1979).

Our results show that the Pekkarinen et al.'s (2015) reindeer‐lichen model is able to describe the changes in lichen biomass with good accuracy when newly estimated lichen wastages are used. This is the first time that a complex dynamic consumer‐resource model for a reindeer‐lichen system, or any other ungulate‐plant system as far as we are aware, has been validated with respect to its ability to describe changes in resource abundance. Our work shows that a detailed description of the system that takes into account all relevant factors can be made accurate enough to be used for describing and predicting changes in the system and also for studying sustainable management. This requires both the parameters and model structure to be in line with the real system. We showed that the model without relevant extensions was unable to describe the system with good accuracy, even with the estimated wastage parameters.

In the cross‐validation method, the data used for the validation run are not used in corresponding parameter estimation, thus allowing the same data to be used in both parameter estimation and model validation. However, the best option for model validation would be to have new independent data, but unfortunately this is often not possible when studying large, complex systems. This has also been the case in our study, as our sample size is only 20 herding districts. Thus, a study with new datasets would further increase the reliability of the validation and the quantitative predictions by the model. However, the fact that all predetermined extensions (except supplementary feeding) increased the model fit to the data indicates that the structure and various components of the model represent the system well.

Pekkarinen et al. (2015) used the reindeer‐lichen model to compute the optimal solutions for the system. In our study, we used the same model with new wastage parameters. We found that the qualitative results are similar with various wastages, but there are important quantitative differences. Lichen biomass, size of reindeer population, and net revenues in the optimal steady state are lower with the new wastage values. We also found that when the wastage relative to the intake rate increases as a function of lichen biomass, it favors the use of intensive supplementary feeding in the optimal steady state. Pekkarinen et al. (2015) assumed that wastage during the summer period is twice the intake rate and found that a closed pasture rotation system should be used to prevent the trampling of winter pasture resources during summer. In our study, we found that the wastage during summer is three to five times higher than those used by Pekkarinen et al. (2015). Thus, reindeer management based on natural pastures benefits from seasonal pasture rotation even more than previously expected. This also suggests that without pasture rotation it is more likely that the optimal solution includes the use of supplementary food in the long‐term steady state. In Sweden and Norway, a pasture rotation system is used in most cases, leading to summertime trampling to be a problem mainly in Finland. However, trampling has also caused the deterioration of lichen pastures in other Nordic countries when reindeer have been introduced to islands, fences have blocked the movements of reindeer, or reindeer density has increased to a very high level (Hansen et al., 2007; Moen & Danell, 2003).

Overall the solutions for optimal management do not differ much when a constant share of wintertime wastage is changed from 0.3 used by Pekkarinen et al. (2015) to 0.5 found in our study. If the wastage relative to intake rate is described with an increasing linear function of lichen biomass, the differences in the results are larger. However, qualitatively (how well the model analyzes describe various effects and mechanisms in the system) the results remain similar and both constant and linear wastage works relatively well in model validation. Furthermore, it should be recognized that the actual wastages are probably slightly lower than those found in our study because other factors besides reindeer consumption, infrastructure, and heavy metals might also affect the reduction of lichen biomass. The actual growth rate of lichens might also be lower than used in our model if grazing and trampling additionally reduce it. For the same reason and because of the high lichen biomasses in districts 4, 5, and 6 in year 1995 (see Table 1) strongly affect the solutions and favor linear wastage compared to constant wastage, it is possible that the dependence of wastage on lichen biomass could be smoother than that found in our study and thus closer to constant wastage.

We used the validated model to compare actual reindeer numbers and lichen biomasses to optimal management solutions. We found that with the linear wastage function the optimal lichen biomasses in steady‐state solutions are lower than those found in previous optimization studies. However, it appears that these optimal lichen biomasses were still higher than in the present situation in most of the herding districts studied. Accordingly, the number of reindeer was also clearly higher in all except two herding districts. This shows that lichen biomasses may still continue to decrease in most of the districts at current reindeer levels and pasture states. This would lead to reindeer management having to increasingly rely on supplementary feeding. From the herders viewpoint this might still be the optimal solution if they receive government subsidies, prefer a higher interest rate and/or have access to low‐cost supplementary food (see Figure 5). Herders might also keep larger herds as a risk reducing strategy in a stochastic environment (Næss & Bårdsen, 2010) or to gain social status within the community (Johannesen & Skonhoft, 2011). However, the long‐term changes in species composition and for the entire pasture ecosystem will be difficult to predict.

Mysterud (2006) suggests a qualitative grading system for the assessment of overgrazing until methods for quantitatively measuring overgrazing are developed. In our study, we used a validated model with estimated wastages to compare the optimal steady‐state results with the current situation in 20 herding districts, and found that reindeer numbers are in many cases clearly above the steady‐state situation. Thus, our approach can be used as a more quantitative way of measuring overgrazing of lichen pastures. The method presented in this study for the estimation of wastage and evaluation of overgrazing can also be used for other similar ungulate‐pasture systems if the resource biomass is strongly affected by ungulate grazing. However, this is not always the case as some plants tolerate or even benefit from grazing (Hobbs, 1996), and thus, the effects of grazing and trampling do not necessarily show as reduction of plant biomass in following years (Kohler, Gillet, Gobat, & Buttler, 2004). In addition, a complex model for estimating wastage and assessing overgrazing requires detailed data of population density and pasture conditions. Such data are difficult to gather for wild ungulates, but bookkeeping is often kept on at least the number of animals for semi‐domesticated and domesticated animals. Still, even data on the number of animals are not always precise. We observed that a certain part of the reindeer population is not counted, possibly because all reindeer are not found during the gatherings. As far as we know, this was the first time that the number of uncounted reindeer has been assessed and included in an analysis. In addition to accurate data on population densities and pasture conditions, the model must be detailed enough to take into account all relevant factors of the system, if this approach is to be used for quantifying overgrazing. Otherwise the model's ability to describe the impact of herbivores on plant communities remains low, even if the wastage is estimated using detailed data and optimization.

## 5 CONCLUSIONS

Our study uses detailed data to parameterize and validate a complex reindeer‐lichen system model. Validation can never prove a model right, but it can help show how it relates to the measured data and represents the problem entity (Brown & Kulasiri, 1996). We found that the Pekkarinen et al. (2015) model can be used relatively accurately for studying actual reindeer‐lichen management systems in northernmost Finland and that their results are well connected to the empirical data. We also show that an overly simplistic model cannot take into account all relevant factors in the system. For example, models for a reindeer‐lichen system should be able to describe districts with and without pasture rotation systems.

Our study presents the first data‐based estimate of a lichen wastage parameter used in reindeer‐lichen models. Wastage by trampling can affect lichen biomass even more than lichen intake; thus, the effects of wastage are as important as other effects of herbivory. Although lichens are probably one of the food resources least tolerant of herbivore grazing and trampling (Mysterud, 2006), the effects of wastage should always be quantified when any herbivore–plant system is studied with complex models. This is especially important with larger ungulates that have a greater ecological impact because of their large hoof size and long daily walking distances (Cumming & Cumming, 2003; cf. Hobbs & Searle, 2005). However, empirical studies that quantify wastage parameters are extremely rare and difficult to directly adjust to models for ungulate‐plant systems. We show how data and a plant–herbivore model with a detailed description of resource intake can be used for estimating wastage levels. Whether or not this approach is suitable for other ungulate‐pasture systems depends highly on plant species tolerance for consumption and trampling. Empirical field research of wastage and trampling is certainly still required.

## CONFLICT OF INTEREST

None declared.

## AUTHORS CONTRIBUTION

Antti‐Juhani Pekkarinen, Jouko Kumpula, and Olli Tahvonen conceived the ideas and designed methodology; Jouko Kumpula and Antti‐Juhani Pekkarinen collected the data; Antti‐Juhani Pekkarinen analyzed the data and led the writing of the manuscript. All authors contributed critically to the drafts and gave final approval for publication.

## Appendix 1 Reindeer numbers in 20 northernmost herding districts (HD) in Finland during 1995–2007

### 1.1

**Table:**

HD	Reindeer	1995	1996	1997	1998	1999	2000	2001	2002	2003	2004	2005	2006	2007	Average
1	Reindeer after slaghtering	7,740	7,577	7,194	7,065	6,746	6,899	7,019	6,328	6,308	6,803	6,255	6,512	6,646	6,853
	Adult before next slaughtering	7,325	7,722	6,872	6,595	6,550	7,481	7,100	6,311	6,375	6,815	6,521	7,000	6,866	6,887
	Calves before next slaughtering	4,014	3,932	3,808	4,835	3,097	3,234	4,590	4,911	5,085	5,096	4,686	4,896	4,763	4,381
2	Reindeer after slaghtering	6,440	6,213	6,225	6,039	6,072	6,263	5,459	5,252	5,456	5,583	5,229	5,684	5,711	5,817
	Adult before next slaughtering	6,155	6,163	6,385	6,214	6,061	6,424	5,685	5,563	5,721	5,729	5,771	5,692	5,898	5,959
	Calves before next slaughtering	3,769	2,263	3,475	3,512	3,260	1,938	3,959	4,037	3,974	3,845	3,602	3,961	4,194	3,522
3	Reindeer after slaghtering	3,515	3,363	3,188	3,367	3,512	2,726	3,510	3,457	3,721	3,569	3,383	3,572	3,408	3,407
	Adult before next slaughtering	3,407	2,980	3,075	3,419	2,867	3,184	3,352	3,488	3,718	3,395	3,254	3,378	3,241	3,289
	Calves before next slaughtering	1,802	1,256	1,295	1,307	1,587	789	1,737	1,702	1,985	2,096	1,923	1,807	1,497	1,599
4	Reindeer after slaghtering	6,333	5,976	5,913	5,915	6,349	5,395	5,449	4,836	5,091	5,169	5,248	5,068	5,294	5,541
	Adult before next slaughtering	6,568	5,690	5,936	6,403	6,341	5,743	5,250	5,120	5,384	5,938	5,798	5,792	5,545	5,808
	Calves before next slaughtering	3,405	2,902	2,797	3,285	3,167	2,682	3,643	3,197	3,031	2,709	3,206	2,967	2,582	3,044
5	Reindeer after slaghtering	2,220	2,144	2,322	2,397	2,566	2,201	2,438	2,527	2,904	2,867	2,975	2,947	3,079	2,584
	Adult before next slaughtering	2,045	2,085	2,227	2,454	2,207	2,405	2,446	2,587	2,850	2,815	2,798	2,928	2,995	2,526
	Calves before next slaughtering	892	820	1,066	1,094	1,139	870	1,164	1,182	1,274	1,378	1,482	1,588	1,517	1,190
6	Reindeer after slaghtering	1,723	1,317	1,732	1,168	1,492	1,336	1,718	1,336	1,580	1,615	1,073	1,244	1,370	1,439
	Adult before next slaughtering	1,327	1,419	1,325	1,574	1,412	1,660	1,588	1,563	1,970	1,187	1,595	1,609	1,271	1,500
	Calves before next slaughtering	976	315	752	552	746	479	629	682	810	884	606	777	597	677
7	Reindeer after slaghtering	5,699	5,457	4,930	5,638	6,246	5,105	5,065	5,297	5,604	6,140	5,713	5,629	5,752	5,560
	Adult before next slaughtering	5,711	5,227	5,688	6,661	6,409	5,233	5,665	6,262	6,516	7,244	6,343	6,181	6,251	6,107
	Calves before next slaughtering	3,729	2,924	2,907	3,015	3,636	2,913	3,011	2,907	3,238	3,656	3,820	3,555	3,173	3,268
8	Reindeer after slaghtering	5,963	5,749	5,566	5,660	5,455	5,249	5,334	4,889	4,943	5,525	5,489	5,192	5,535	5,427
	Adult before next slaughtering	6,478	5,921	6,186	6,262	5,797	5,962	5,535	5,512	5,981	6,099	5,548	6,314	5,860	5,958
	Calves before next slaughtering	3,091	2,234	1,736	2,175	2,560	1,979	2,788	2,277	2,403	2,573	2,273	2,261	2,285	2,357
9	Reindeer after slaghtering	9,046	8,910	8,364	8,283	8,141	6,591	7,417	7,275	7,322	7,452	7,786	7,585	7,347	7,809
	Adult before next slaughtering	9,559	7,750	7,928	7,981	6,512	7,238	7,189	7,439	7,956	8,818	8,518	7,461	7,348	7,823
	Calves before next slaughtering	4,322	4,114	2,502	3,726	3,302	1,710	3,177	2,910	3,131	3,430	2,861	4,460	3,186	3,295
10	Reindeer after slaghtering	7,977	7,994	7,490	7,223	7,478	6,405	7,036	7,257	6,402	7,345	6,653	6,781	7,074	7,163
	Adult before next slaughtering	8,640	7,651	6,902	7,728	6,242	7,008	7,273	6,877	6938	7,136	6,669	6,919	6,664	7,127
	Calves before next slaughtering	4,043	4,375	2,785	4,254	3,106	2,216	3,967	4,291	4,568	4,198	3,554	3,861	2,520	3,672
11	Reindeer after slaghtering	8,693	8,064	6,925	7,301	7,263	6,827	7,649	8,242	9,559	10,178	8,271	10,367	9,179	8,348
	Adult before next slaughtering	7,726	5,623	5,363	5,586	5,098	5,642	6,081	6,759	8,947	8,211	8,854	8,712	8,118	6,978
	Calves before next slaughtering	2,674	2,735	2,210	2,914	2,553	2,471	2,990	3,219	3,762	5,462	4,278	5,241	4,940	3,496
12	Reindeer after slaghtering	8,364	9,157	9,439	8,639	8,439	9,401	10,586	9,962	11,526	11,290	11,698	9,805	10,615	9,917
	Adult before next slaughtering	7,240	7,489	6,938	6,452	6,800	7,416	8,650	9,068	10,155	10,592	10,223	10,912	10,741	8,667
	Calves before next slaughtering	2,860	3,140	3,207	3,343	2,824	3,009	3,944	4,853	5,434	6,599	6,494	6,610	5,996	4,486
13	Reindeer after slaghtering	7,286	6,212	4,658	4,771	5,706	4,936	5,077	5,702	5,556	5,867	5,926	5,988	5,998	5,668
	Adult before next slaughtering	6,008	4,095	4,374	4,854	4,607	4,426	4,480	4,770	5,307	5,555	6,096	6,117	5,717	5,108
	Calves before next slaughtering	1,749	1,170	1,935	2,121	2,308	1,988	2,162	2,170	2,469	3,168	3,164	3,178	3,076	2,358
14	Reindeer after slaghtering	3,394	3,206	2,431	2,356	2,766	2,764	2,688	3,276	3,445	3,785	3,320	3,424	3,449	3,100
	Adult before next slaughtering	2,938	2,253	1,870	2,175	2,315	2,201	2,630	2,984	3,466	3,548	3,344	3,547	3,650	2,840
	Calves before next slaughtering	772	721	969	886	969	988	1,028	1,365	1,709	1,883	1,883	2,188	1,863	1,325
15	Reindeer after slaghtering	5,390	4,641	4,129	4,377	4,889	4,194	4,772	5,062	5,806	5,770	5,425	5,374	5,645	5,036
	Adult before next slaughtering	4,777	3,721	4,006	4,445	4,176	4,479	4,722	5,230	5,767	5,821	5,496	5,860	5,149	4,896
	Calves before next slaughtering	2,015	820	1,809	2,080	2,045	1,922	2,047	2,238	2,617	2,380	2,432	2,280	2,463	2,088
16	Reindeer after slaghtering	5,838	4,520	4,256	4,810	5,231	4,779	5,161	5,108	5,330	6,060	5,411	5,258	5,216	5,152
	Adult before next slaughtering	4,792	4,068	4,678	5,041	4,867	5,052	5,083	5,068	5,964	5,977	5,691	5,240	5,144	5,128
	Calves before next slaughtering	3,023	1,653	2,357	2,753	2,670	2,529	2,656	2,622	2,845	2,883	3,027	3,205	2,922	2,703
17	Reindeer after slaghtering	5,665	5,529	5,911	5,884	5,960	5,473	6,065	5,876	5,999	6,202	5,815	5,206	5,382	5,767
	Adult before next slaughtering	5,247	5,823	6,071	5,975	5,662	5,857	6,117	5,904	6,206	6,286	5,550	5,348	5,308	5,796
	Calves before next slaughtering	3,026	1,999	3,492	3,709	3,618	3,710	3,784	3,830	4,237	4,352	4,155	3,690	3,448	3,619
18	Reindeer after slaghtering	8,997	8,602	7,444	7,371	8,304	6,143	8,521	7,176	7,012	8,357	6,437	7,121	7,826	7,639
	Adult before next slaughtering	8,274	7,486	7,604	8,061	6,395	7,445	8,225	6,840	8,719	7,870	7,377	7,731	7,814	7,680
	Calves before next slaughtering	4,819	1,953	3,164	3,634	3,008	2,152	3,856	3,308	3,684	3,841	3,685	3,871	3,867	3,449
19	Reindeer after slaghtering	11,803	11,092	11,316	11,198	12,505	11,428	13,225	12,736	11,985	12,652	11,776	11,797	12,148	11,974
	Adult before next slaughtering	10,266	11,311	11,648	12,657	12,170	13,507	14,428	13,326	13,747	12,759	11,911	12,275	12,008	12,463
	Calves before next slaughtering	8,771	3,748	6,364	6,275	6,265	6,451	8,015	8,575	9,515	9,143	8,826	8,162	7,499	7,508
20	Reindeer after slaghtering	5,242	5,424	4,805	4,820	4,669	4,523	4,860	4,666	4,638	4,773	5,144	4,491	4,509	4,813
	Adult before next slaughtering	5,580	5,209	4,868	4,737	4,779	5,055	4,966	4,731	4,926	5,311	4,871	4,501	4,664	4,938
	Calves before next slaughtering	2,936	1,593	2,789	2,608	2,794	2,219	2,429	2,794	2,833	2,486	2,958	2,879	1,991	2,562

## Appendix 2 The mortality and uncounted reindeer during 1995–2007 in all study areas

### 2.1

**Table:**

HD	Number of reindeer	1995	1996	1997	1998	1999	2000	2001	2002	2003	2004	2005	2006	2007	Average
1	After slaghtering	7,740	7,577	7,194	7,065	6,746	6,899	7,019	6,328	6,308	6,803	6,255	6,512	6,646	6,853
	Before nex slaughtering	7,325	7,722	6,872	6,595	6,550	7,481	7,100	6,311	6,375	6,815	6,521	7,000	6,866	6,887
	Mortality due to traffic & predators (%)	3	5	1	3	5	2	0	0	1	1	1	2	2	2
	Uncounted (%)	0	7	0	0	2	10	2	0	2	1	5	9	5	3
2	After slaghtering	6,440	6,213	6,225	6,039	6,072	6,263	5,459	5,252	5,456	5,583	5,229	5,684	5,711	5,817
	Before next slaughtering	6,155	6,163	6,385	6,214	6,061	6,424	5,685	5,563	5,721	5,729	5,771	5,692	5,898	5,959
	Mortality due to traffic & predators (%)	2	2	2	2	5	1	0	0	0	1	1	2	1	1
	Uncounted (%)	0	1	4	5	5	4	4	6	5	3	10	2	4	4
3	After slaghtering	3,515	3,363	3,188	3,367	3,512	2,726	3,510	3,457	3,721	3,569	3,383	3,572	3,408	3,407
	Before next slaughtering	3,407	2,980	3,075	3,419	2,867	3,184	3,352	3,488	3,718	3,395	3,254	3,378	3,241	3,289
	Mortality due to traffic & predators (%)	0	0	0	0	0	0	0	0	1	1	1	2	3	1
	Uncounted (%)	0	0	0	2	0	14	0	1	1	0	0	0	0	1
4	After slaghtering	6,333	5,976	5,913	5,915	6,349	5,395	5,449	4,836	5,091	5,169	5,248	5,068	5,294	5,541
	Before next slaughtering	6,568	5,690	5,936	6,403	6,341	5,743	5,250	5,120	5,384	5,938	5,798	5,792	5,545	5,808
	Mortality due to traffic & predators (%)	1	1	3	1	1	1	1	1	2	3	2	3	5	2
	Uncounted (%)	5	0	3	9	1	7	0	6	7	15	11	15	9	7
5	After slaghtering	2,220	2,144	2,322	2,397	2,566	2,201	2,438	2,527	2,904	2,867	2,975	2,947	3,079	2,584
	Before next slaughtering	2,045	2,085	2,227	2,454	2,207	2,405	2,446	2,587	2,850	2,815	2,798	2,928	2,995	2,526
	Mortality due to traffic & predators (%)	1	1	0	0	1	0	0	0	1	1	1	1	1	1
	Uncounted (%)	0	0	0	3	0	9	1	3	0	0	0	0	0	1
6	After slaghtering	1,723	1,317	1,732	1,168	1,492	1,336	1,718	1,336	1,580	1,615	1,073	1,244	1,370	1,439
	Before next slaughtering	1,327	1,419	1,325	1,574	1,412	1,660	1,588	1,563	1,970	1,187	1,595	1,609	1,271	1,500
	Mortality due to traffic & predators (%)	1	1	1	1	1	2	3	0	1	1	1	1	3	1
	Uncounted (%)	0	8	0	27	0	22	0	15	20	0	33	23	0	11
7	After slaghtering	5,699	5,457	4,930	5,638	6,246	5,105	5,065	5,297	5,604	6,140	5,713	5,629	5,752	5,560
	Before next slaughtering	5,711	5,227	5,688	6,661	6,409	5,233	5,665	6,262	6,516	7,244	6,343	6,181	6,251	6,107
	Mortality due to traffic & predators (%)	3	3	6	2	3	3	2	2	4	4	3	3	4	3
	Uncounted (%)	4	0	18	17	6	5	13	17	18	18	12	12	12	12
8	After slaghtering	5,963	5,749	5,566	5,660	5,455	5,249	5,334	4,889	4,943	5,525	5,489	5,192	5,535	5,427
	Before next slaughtering	6,478	5,921	6,186	6,262	5,797	5,962	5,535	5,512	5,981	6,099	5,548	6,314	5,860	5,958
	Mortality due to traffic & predators (%)	2	3	1	3	3	1	2	2	2	3	3	4	3	2
	Uncounted (%)	10	6	11	12	9	13	5	13	19	12	4	21	9	11
9	After slaghtering	9,046	8,910	8,364	8,283	8,141	6,591	7,417	7,275	7,322	7,452	7,786	7,585	7,347	7,809
	Before next slaughtering	9,559	7,750	7,928	7,981	6,512	7,238	7,189	7,439	7,956	8,818	8,518	7,461	7,348	7,823
	Mortality due to traffic & predators (%)	2	3	4	4	6	4	3	3	4	5	4	5	4	4
	Uncounted (%)	7	0	0	0	0	13	0	5	12	19	12	3	4	6
10	After slaghtering	7,977	7,994	7,490	7,223	7,478	6,405	7,036	7,257	6,402	7,345	6,653	6,781	7,074	7,163
	Before next slaughtering	8,640	7,651	6,902	7,728	6,242	7,008	7,273	6,877	6,938	7,136	6,669	6,919	6,664	7,127
	Mortality due to traffic & predators (%)	6	7	6	4	5	5	1	1	3	6	4	9	3	5
	Uncounted (%)	13	2	0	11	0	13	5	0	11	3	5	10	0	6
11	After slaghtering	8,693	8,064	6,925	7,301	7,263	6,827	7,649	8,242	9,559	10,178	8,271	10,367	9,179	8,348
	Before next slaughtering	7,726	5,623	5,363	5,586	5,098	5,642	6,081	6,759	8,947	8,211	8,854	8,712	8,118	6,978
	Mortality due to traffic & predators (%)	4	7	4	5	4	4	3	4	4	6	7	6	6	5
	Uncounted (%)	0	0	0	0	0	0	0	0	0	0	13	0	0	1
12	After slaghtering	8,364	9,157	9,439	8,639	8,439	9,401	10,586	9,962	11,526	11,290	11,698	9,805	10,615	9,917
	Before next slaughtering	7,240	7,489	6,938	6,452	6,800	7,416	8,650	9,068	10,155	10,592	10,223	10,912	10,741	8,667
	Mortality due to traffic & predators (%)	9	8	3	8	9	6	3	3	3	6	7	8	6	6
	Uncounted (%)	0	0	0	0	0	0	0	0	0	0	0	17	7	2
13	After slaghtering	7,286	6,212	4,658	4,771	5,706	4,936	5,077	5,702	5,556	5,867	5,926	5,988	5,998	5,668
	Before next slaughtering	6,008	4,095	4,374	4,854	4,607	4,426	4,480	4,770	5,307	5,555	6,096	6,117	5,717	5,108
	Mortality due to traffic & predators (%)	2	1	2	3	3	3	2	4	5	5	5	6	8	4
	Uncounted (%)	0	0	0	5	0	0	0	0	1	0	8	8	3	2
14	After slaghtering	3,394	3,206	2,431	2,356	2,766	2,764	2,688	3,276	3,445	3,785	3,320	3,424	3,449	3,100
	Before next slaughtering	2,938	2,253	1,870	2,175	2,315	2,201	2,630	2,984	3,466	3,548	3,344	3,547	3,650	2,840
	Mortality due to traffic & predators (%)	2	2	1	2	2	1	0	1	2	2	2	2	1	2
	Uncounted (%)	0	0	0	0	0	0	0	0	3	0	3	5	7	1
15	After slaghtering	5,390	4,641	4,129	4,377	4,889	4,194	4,772	5,062	5,806	5,770	5,425	5,374	5,645	5,036
	Before next slaughtering	4,777	3,721	4,006	4,445	4,176	4,479	4,722	5,230	5,767	5,821	5,496	5,860	5,149	4,896
	Mortality due to traffic & predators (%)	2	2	1	2	1	2	1	1	1	1	1	5	2	2
	Uncounted (%)	0	0	0	3	0	8	0	4	0	2	3	13	0	3
16	After slaghtering	5,838	4,520	4,256	4,810	5,231	4,779	5,161	5,108	5,330	6,060	5,411	5,258	5,216	5,152
	Before next slaughtering	4,792	4,068	4,678	5,041	4,867	5,052	5,083	5,068	5,964	5,977	5,691	5,240	5,144	5,128
	Mortality due to traffic & predators (%)	2	1	2	2	2	3	1	2	2	2	2	6	2	2
	Uncounted (%)	0	0	11	7	0	8	0	1	12	1	7	5	1	4
17	After slaghtering	5,665	5,529	5,911	5,884	5,960	5,473	6,065	5,876	5,999	6,202	5,815	5,206	5,382	5,767
	Before next slaughtering	5,247	5,823	6,071	5,975	5,662	5,857	6,117	5,904	6,206	6,286	5,550	5,348	5,308	5,796
	Mortality due to traffic & predators (%)	2	2	2	4	4	4	2	3	2	3	3	2	2	3
	Uncounted (%)	0	7	5	5	0	10	3	3	6	4	0	5	1	4
18	After slaghtering	8,997	8,602	7,444	7,371	8,304	6,143	8,521	7,176	7,012	8,357	6,437	7,121	7,826	7,639
	Before next slaughtering	8,274	7,486	7,604	8,061	6,395	7,445	8,225	6,840	8,719	7,870	7,377	7,731	7,814	7,680
	Mortality due to traffic & predators (%)	2	3	4	5	4	5	2	3	3	5	7	9	4	4
	Uncounted (%)	0	0	6	13	0	22	0	0	22	0	19	16	4	8
19	After slaghtering	11,803	11,092	11,316	11,198	12,505	11,428	13,225	12,736	11,985	12,652	11,776	11,797	12,148	11,974
	Before next slaughtering	10,266	11,311	11,648	12,657	12,170	13,507	14,428	13,326	13,747	12,759	11,911	12,275	12,008	12,463
	Mortality due to traffic & predators (%)	1	1	1	2	1	1	1	1	1	2	1	2	2	1
	Uncounted (%)	0	3	4	13	0	16	9	5	14	2	2	6	1	6
20	After slaghtering	5,242	5,424	4,805	4,820	4,669	4,523	4,860	4,666	4,638	4,773	5,144	4,491	4,509	4,813
	Before next slaughtering	5,580	5,209	4,868	4,737	4,779	5,055	4,966	4,731	4,926	5,311	4,871	4,501	4,664	4,938
	Mortality due to traffic & predators (%)	3	2	1	2	2	2	3	2	2	2	4	8	8	3
	Uncounted (%)	9	0	3	0	4	13	5	3	8	12	0	8	11	6
